# Associations between a Universal Free Breakfast Policy and School Breakfast Program Participation, School Attendance, and Weight Status: A District-Wide Analysis

**DOI:** 10.3390/ijerph19073749

**Published:** 2022-03-22

**Authors:** Sally Lawrence Bullock, Spring Dawson-McClure, Kimberly Parker Truesdale, Dianne Stanton Ward, Allison E. Aiello, Alice S. Ammerman

**Affiliations:** 1 Department of Public Health, Davidson College, P.O. Box 7135, Davidson, NC 28035, USA; 2 Department of Population Health, NYU Grossman School of Medicine, 227 East 30th Street Seventh Floor, New York, NY 10016, USA; spring.dawson-mcclure@nyulangone.org; 3 Department of Nutrition, Gillings School of Global Public Health, The University of North Carolina at Chapel Hill, 170 Rosenau Hall, CB #7461, Chapel Hill, NC 27599, USA; kim_truesdale@unc.edu (K.P.T.); dsward@email.unc.edu (D.S.W.); alice_ammerman@unc.edu (A.S.A.); 4 Department of Epidemiology, Gillings School of Global Public Health, The University of North Carolina at Chapel Hill, 170 Rosenau Hall, CB #7400, Chapel Hill, NC 27599, USA; aaiello@unc.edu

**Keywords:** School Breakfast Program, children, schools, nutrition policy, program evaluation

## Abstract

Breakfast consumption among youth is associated with improved diet quality, weight, cognition, and behavior. However, not all youth in the United States consume breakfast. Participation in the School Breakfast Program (SBP) is also low relative to the lunch program. Universal free breakfast (UFB) policies have been implemented to increase breakfast participation by reducing cost and stigma associated with the SBP. This study examined whether a UFB policy implemented in a school district in the Southeast US was associated with changes in breakfast participation, school attendance, and student weight. A longitudinal study of secondary data was conducted, and a mixed modeling approach was used to assess patterns of change in SBP participation. General linear models were used to assess attendance and student weight change. On average, across schools in the district, there was an increase in breakfast participation of 4.1 percentage points following the implementation of the policy. The change in breakfast participation in schools differed by the percent of students in the school who received school meals for free or at a reduced price, the percent of students of color, and the grade level of the school. Increases in SBP participation were not associated with significant changes in attendance or weight. UFB policies may be effective in increasing participation in the SBP.

## 1. Introduction

In the United States, almost 20 percent of children and adolescents between the ages of 2 and 19 do not consume breakfast [1]. In addition, there is a decreasing trend in breakfast consumption with age [2], and children and adolescents from families with lower incomes are less likely to consume breakfast than those from families with higher incomes [1]. These trends in breakfast consumption are potentially problematic, as eating breakfast may lead to improved nutrient profiles, better weight status, and improved cognitive function and behavior among children [3,4,5,6,7,8,9,10]. The School Breakfast Program (SBP), which became a permanent entitlement program in the United States in 1975, was created to ensure that school-aged children had access to a meal to start the school day [11]. Across the country, the SBP is available in approximately 94% of schools that serve lunch [12], and about 95% of all public schools in the United States offer the National School Lunch Program [13]. Some students are eligible to receive free or reduced-price (FRP) meals based on their family income [12]. Despite the high availability, participation in the SBP is much lower than participation in the National School Lunch Program [12].

Many factors may influence participation in the SBP. Quality, variety, taste, and appearance of the food have all been cited by students as key factors in their decision to participate in the school meal programs [14,15,16]. The price of meals may also be a deterrent for some students [17,18], especially for those paying for reduced or full-price meals. School schedules and the time and convenience of accessing school breakfast may also be a factor in students’ decision to participate [17,19,20]. Several studies have also found that participation is associated with certain demographic characteristics—African American students, males, younger students, and students living in rural areas are more likely to participate [15,21,22,23]. Stigma has also been identified as a particularly important influence on participation rates and may explain some of the differences in participation by various demographic characteristics [18,20,24]. The stigma associated with school breakfast consumption may stem from the perception that only “poor kids” eat school meals [24].

Alternative breakfast service models, like universal free breakfast, breakfast in the classroom, grab and go, and second chance breakfast, have been implemented in schools to overcome barriers and increase participation in the SBP [25]. Implementing universal free breakfast (UFB), where all students receive free school breakfast, may help to reduce barriers around cost and the stigma associated with breakfast participation [26]. Studies of UFB programs and policies indicate that they are associated with an increase in SBP participation [27,28,29,30,31,32,33,34,35]. However, the extent to which the increase in participation in the SBP leads to improvements in academic and health-related indicators, such as school attendance and weight status, is not clear. In addition, the majority of studies of UFB initiatives have focused on primary schools and have not included middle and high schools. The purpose of this observational study was to determine (1) whether a district-wide UFB policy implemented in a large urban school district in the Southeast United States was associated with an increase in school-level breakfast participation, and (2) whether schools with an increase in breakfast participation following the implementation of the UFB policy were more likely to maintain or improve student attendance levels and student weight status than schools that did not have an increase in participation.

## 2. Materials and Methods

### 2.1. Setting

The large urban school district that is the focus of this study implemented its universal free breakfast (UFB) policy in all schools in the district during the 2013–2014 school year. As of the 2015–2016 school year, the school district had over 146,000 students enrolled in 168 schools, including 91 elementary schools, 30 middle schools, 31 high schools, and 16 “other” schools (Pre-K-8, K-8, K-12, 6-12, and alternative schools). Approximately 37% of the students in the district are African American, 28% are Hispanic, 25% are White, 7% are Asian American, 3% are multiracial, and less than 1% are Native American or Pacific Islander. Nearly 30% of the student body in the district are automatically certified for free school meals based on their participation in federal benefits programs. However, the percent of students that are automatically certified ranges from 0% to over 70% at individual schools in the district. There are 85 Title 1 schools in the district (schools in which at least 40% of students are from low-income families).

All school levels were included in the analyses. However, alternative schools (schools designed to educate students who have not been successful in traditional schools because of behavioral, disciplinary, and/or safety concerns), Pre-K only schools, and schools that serve only students with special needs were excluded given their specialized nature and unique student population. Schools that did not have complete data for the 2012–2013 and/or 2013–2014 school years were also excluded. As a result, 150 schools were included in the analyses.

### 2.2. Variables and Data Sources

#### 2.2.1. Outcome Variables

The outcome variables for the analyses included school-level participation in the SBP, change in attendance, and change in student weight status. School-level participation in the SBP was calculated by dividing the total number of school breakfasts served during a school year by the product of the average daily membership and the number of days that year that breakfasts were served. The average daily membership is equal to the average number of students enrolled within a school each day during the school year. The official average daily membership for each school for each year was available on the state Department of Public Instruction website [36]. Other data needed to calculate SBP participation were provided directly by the school district.

The school-level change in attendance variable was calculated by subtracting attendance for 2012–2013 (before the policy) from attendance for 2013–2014 (after the policy). School-level attendance for each year was calculated by dividing the total number of full-day student absences by the total number of days in membership. Total absences and days in membership were calculated using student-level attendance data provided by the Institute for Social Capital [37]. Data for students in grades other than kindergarten through 12th grade were excluded (Pre-K, 5th year of high school, etc.). Change in unexcused absences and days tardy were also calculated using this method.

School-level changes in the percent of overweight and obese students were calculated using student height and weight data collected annually, and in some cases biannually, by the school district physical education teachers. Students’ BMI-for-age and -sex percentiles were calculated using a SAS Program based on the 2000 Centers for Disease Control and Prevention (CDC) Growth Charts [38]. Students were classified as underweight, healthy weight, overweight, or obese based on the CDC recommended BMI-for-age cutoffs [39]. The number of students in each school in each category was determined, and the percent of students in each category was calculated by dividing the number of students in each category at each school by the total number of students with usable data from each school. Schools without data or with data for only a small percent of their total student population in either the 2012–2013 or 2013–2014 school years were excluded.

#### 2.2.2. Covariates

All covariates for the analyses were selected *a priori.* For the school breakfast participation analysis, variables for school-grade level, percent of students of color, and percent of free or reduced-price (FRP)-eligible students were included in the model. School grade-level information was from the National Center for Education Statistics website [40], and enrollment numbers by race and FRP eligibility were available on the state Department of Public Instruction website [41,42]. Schools were coded as elementary, middle, high, or “other school” (K-8, K-12, 6-12, and 9th grade only). The percent of students of color was calculated by dividing the number of students enrolled in a school who were not classified as “white” by the total number of students enrolled in the school and categorized into three levels: low (≤30%), medium (>30% and ≤70%), and high (>70%). The percent of students of color for 2012–2013 was subtracted from the percent of students of color for 2013–2014 to create another variable, change in percent students of color. The percent of students eligible for FRP meals was calculated by dividing the number of FRP-eligible students in each school by the average daily membership for each school and categorized into three levels: low (≤30%), medium (>30% and ≤70%), and high (>70%). The percent of FRP students for 2012–2013 was subtracted from the percent of FRP students for 2013–2014 to create a change in percent FRP value for each school.

For the change in attendance and percent of students classified as overweight and obese analyses, school-grade level, 2012–2013 breakfast participation rate, percent of students of color, change in percent of students of color, percent FRP, and change in percent of students eligible for FRP were included in the models. In addition, the change in breakfast participation between 2012–2013 and 2013–2014 was also included in the models. The change in breakfast participation was calculated by subtracting school-level breakfast participation for 2012-13 from breakfast participation for 2013–2014.

### 2.3. Statistical Methods

Descriptive information was generated for variables of interest. General linear mixed models were estimated using the maximum likelihood for the breakfast participation analysis. For this analysis, level-1 occasions were nested within level-2 schools, and a piecewise/spline model was estimated with two pieces/slopes. The intercept was at time 0 (the 2006–2007 school year) and there was a breakpoint and a jump/shift in intercept when the policy was implemented after time 6 (2012/2013). The piecewise model with a random intercept, two random linear slopes, and a random jump allowed for the comparison of slopes before and after the implementation of the policy and the immediate shift in the intercept after the policy. Conditional growth models including covariates were then examined using the piecewise model as a baseline. Likelihood ratio tests, Bayesian Information Criterion (BIC), and Akaike Information Criterion (AIC) were used to select the best model for this analysis.

General linear models were estimated to determine associations between changes in breakfast participation and changes in school-level attendance and the percent of students classified as overweight and obese. For each outcome variable, residual normality, linearity, homogeneity of variance, and influential outliers were assessed. Models were run with and without outliers, and no meaningful differences in parameter estimates were observed. All analyses were conducted using SAS version 9.4 [43].

## 3. Results

### 3.1. School Breakfast Participation

For the breakfast participation analysis, a total of 150 schools were included in the dataset per year over nine years for a total of 1306 observations. Demographic data for the schools during the school year before the policy (2012–2013) and the year after the policy was implemented (2013–2014) are provided in Table 1.

#### 3.1.1. Baseline Unconditional Model

**Figure 1:**
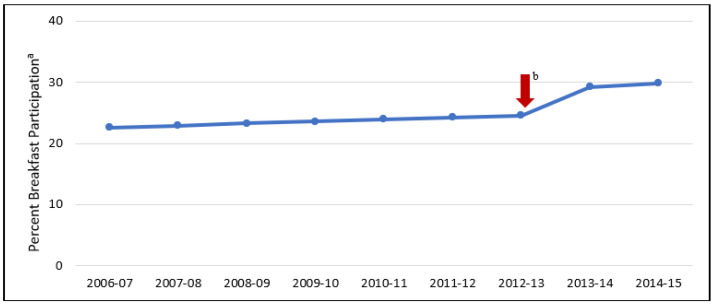
The estimated average percent participation in the National School Breakfast Program before (2006–2007 to 2012–2013) and after (2013–2014 to 2014–2015) the implementation of a districtwide universal free breakfast policy in a large urban school district in the Southeast United States. ^a^ Estimated using an unconditional piecewise general liner mixed model with a random intercept, two random linear slopes, and a random jump. ^b^ The arrow represents the implementation of the 2013 Universal Free Breakfast Policy in the school district.

Figure 1 displays the trajectory of breakfast participation from 2006–2007 to 2014–2015, as estimated by the unconditional model. The intercept estimates an average participation rate of 22.6% (SE = 1.4, *p* < 0.001) during the 2006–2007 school year. The average initial rate of change (slope 1) before the policy was implemented was estimated to be 0.3 percentage points per year (SE = 0.1, *p* = 0.005). The slope after the policy was implemented (slope 2) was estimated to be 0.2 percentage points per year (SE = 0.4, *p* = 0.6). There was a shift or jump in the intercept of 4.1 percentage points (SE = 0.7, *p* < 0.001) immediately after policy implementation (after the 2012–2013 school year).

#### 3.1.2. Final Conditional Model

**Figure 2:**
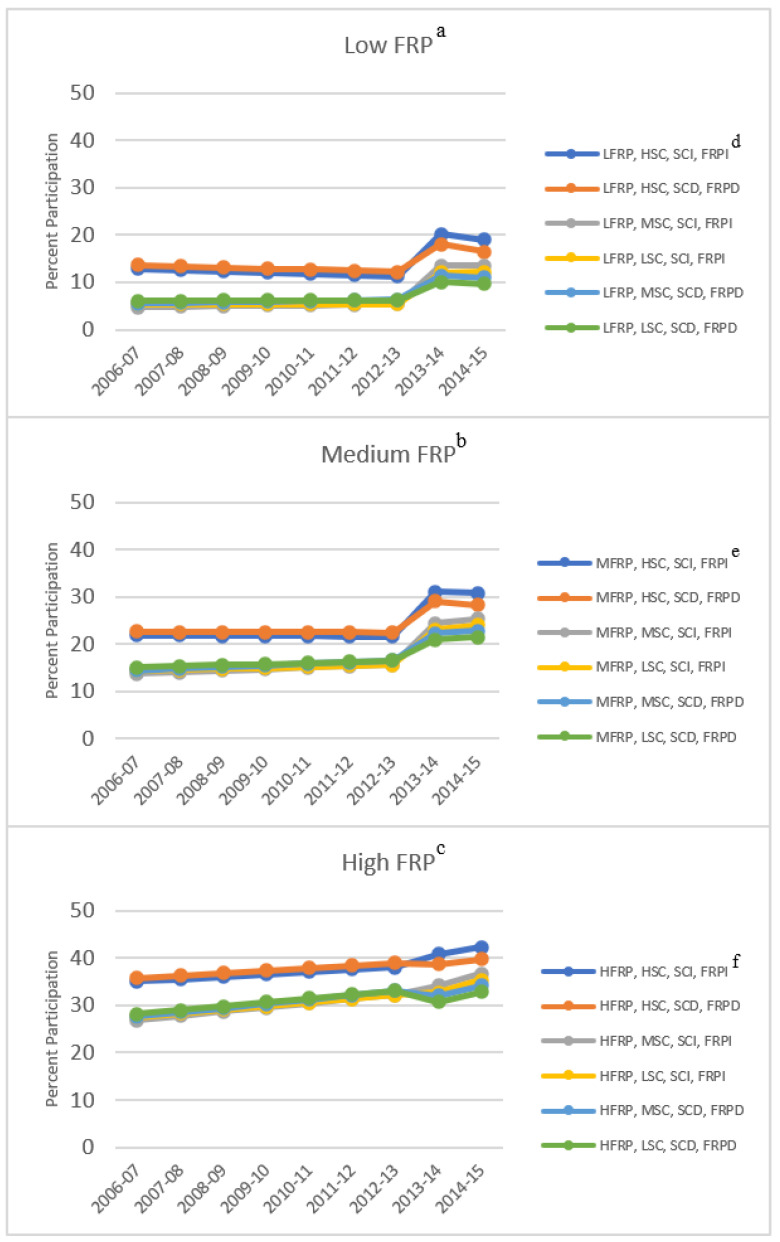
The estimated average percent participation in the National School Breakfast Program before (2006–2007 to 2012–2013) and after (2013–2014 to 2014–2015) the implementation of a districtwide universal free breakfast policy in a large urban school district in the Southeast United States by free and reduced-price (FRP) meal eligibility, percent of students of color, percent change in students of color, and percent change in FRP-eligible students. Estimated using a conditional piecewise general liner mixed model with a random intercept, two random linear slopes, and a random jump. ^a^ Low percent free and reduced-price (LFRP) eligibility is equal to <30% of students eligible for free or reduced-price meals. ^b^ Medium percent free and reduced-price (MFRP) eligibility is equal to ≥30% and <70% of students eligible for free or reduced-price meals. ^c^ High percent free and reduced-price (HFRP) eligibility is equal to ≥70% of students eligible for free or reduced-price meals. ^d,e,f^ Low percent of students of color (LSC) is equal to <30% students of color, medium percent of students of color (MSC) is equal to ≥30% and <70% students of color, high percent of students of color (HSC) is equal to ≥70% students of color, percent change in students of color (decrease in students of color (SCD) = −2%, increase in students of color (SCI) = 2%), percent change in FRP-eligible students (FRP decrease (FRPD) = −2%, FRP Increase (FRPI) = 2%).

The final conditional model included the following school-level covariates: school grade-level, percent students of color, percent change in students of color, percent of FRP-eligible students, and percent change in FRP-eligible students. The model also contained interaction terms for each of these covariates and the two slopes and jump. Results from the final model are summarized below. Figure 2 contains estimated SBP participation rates for school years 2006–2007 to 2014–2015 by FRP eligibility, percent students of color, percent change in students of color, and percent change in FRP-eligible students.

School Grade-Level: Relative to elementary schools, middle schools had significantly lower breakfast participation in 2006–2007 (−7.8 percentage points SE = 1.9, *p* < 0.001) and a significantly less positive change in participation rate from 2012-13 to 2014–2015 (−3.0 SE = 1.1, *p* < 0.01). Similarly, relative to elementary schools, high schools had significantly less positive breakfast participation in 2006–2007 (−17.2 SE = 2.2, *p* < 0.001) and significantly less positive change in participation rate from 2012–2013 to 2014–2015 (−3.1 SE = 1.2, *p* = 0.01). “Other” schools (K-8, K-12, 6-12, and 9th grade only) had significantly higher breakfast participation in 2006–2007 (10.3 SE = 2.6, *p* < 0.001) than elementary schools and significantly less positive change in participation rate from 2012–2013 to 2014–2015 (−4.0 SE = 1.5, *p* < 0.001).

Percent Students of Color: Relative to schools with a high percentage of students of color (≥70%), schools with a medium percentage of students of color (30% ≥ Medium < 70%) had significantly lower breakfast participation in 2006–2007 (−7.0 SE = 2.4, *p* < 0.01), as did schools with a low percentage of students of color (<30%) (−6.7 SE = 3.6, *p* = 0.07). There were no significant effects of the change in the percent of students of color between 2012–2013 and 2013–2014.

Percent FRP Students: Relative to schools with a high percentage of FRP-eligible students (≥70%), schools with a medium percentage of FRP-eligible students (30% ≥ Medium < 70%) had significantly lower breakfast participation in 2006–2007 (−10.7 SE = 2.0, *p* < 0.001) and a significantly greater increase or jump in participation after 2012–2013 (8.7 SE = 2.1, *p* < 0.001). Similarly, relative to schools with a high percentage of FRP-eligible students, schools with a low percentage of FRP-eligible students (<30%) had significantly lower breakfast participation in 2006–2007 (−22.4 SE = 3.2, *p* < 0.001) and a significantly greater increase or jump in participation after 2012–2013 (9.0 SE = 3.2, *p* < 0.01).

### 3.2. School Attendance

Overall, there was an average observed increase in school-level attendance of 0.3 percentage points between the 2012–2013 and 2013–2014 school years (*n* = 150, SD = 0.7). The observed mean increase in attendance for schools that increased SBP participation was 0.3 percentage points (*n* = 122, SD = 0.7) and was 0.5 percentage points (*n* = 28, SD = 0.7) for schools that did not increase SBP participation. Results from a linear regression model controlling for school-grade level, 2012-13 breakfast participation rate, percent of students of color, and percent of students eligible for FRP indicate that for every 1 percentage point increase in breakfast participation between 2012–2013 and 2013–2014, the change in attendance was expected to be non-significantly lower by 0.003 in (SE = 0.01, *p* = 0.7).

Between the 2012–2013 and 2013–2014 school years, there was a 5.5 percentage point increase in the percent of unexcused absences (*n* = 150, SD = 8.6). The mean observed increase in unexcused absences for schools with an increase in SBP participation was 5.9 percentage points (*n* = 122, SD = 9.2) and 3.8 percentage points (*n* = 28, SD = 5.6) for schools that did not have an increase in participation. After controlling for school-grade level, 2013 breakfast participation rate, percent of students of color, and percent of students eligible for FRP, there was no significant association between change in breakfast participation and the change in unexcused absences (0.003 SE = 0.1, *p* = 0.9).

The overall percent of days students were tardy decreased by 0.06 percentage points (*n* = 150, SD = 1.3) between the 2012–2013 and 2013–2014 school years. For schools with an increase in SBP participation, the percent of days students were tardy decreased by 0.13 percentage points (*n* = 122, SD = 1.3), and at schools where SBP participation did not increase, the percent of days students were tardy increased by 0.24 percentage points (*n* = 28, SD = 1.4). Results from a linear regression model, controlling for covariates mentioned above, indicate that for every 1 percentage point increase in breakfast participation between 2012–2013 and 2013–2014, the percent of days that students were tardy was expected to be non-significantly lower by 0.03 percentage points (SE = 0.02, *p* = 0.06).

### 3.3. Percent Students Classified as Overweight or Obese

Between 2012–2013 and 2013–2014, there was an observed increase of 0.2 percentage points (SD = 6.4) in the mean percent of students classified as overweight or obese across the study schools (*n* = 86). Among schools that increased SBP participation, there was an observed increase of 0.2 percentage points (*n* = 68, SD = 6.6) in the mean percent of students classified as overweight or obese, and among schools that did not increase participation, there was an increase of 0.01 percentage points (*n* = 18, SD = 6.1). Results from a linear regression model, controlling for covariates mentioned above, indicate that for every 1 percentage point increase in breakfast participation between 2012–2013 and 2013–2014, there was a non-significant increase of 0.03 percentage points in the percent of students classified as overweight or obese (SE = 0.1, *p* = 0.4).

## 4. Discussion

On average, across schools included in the study, there was an immediate uptick in participation right after the UFB policy implementation. However, the rate of increase in participation per year was greater before implementation than in the school year after the policy was implemented. These changes differed among schools according to levels of FRP-eligible students, students of color, and school grade level. Before implementation, schools with lower percentages of FRP-eligible students and students of color had lower breakfast participation than schools with higher percentages. Conversely, the immediate jump in participation after implementation was greater for schools with lower percentages of FRP-eligible students than schools with higher percentages. Middle and high schools also had significantly lower participation rates than elementary schools before the policy and a significantly lower rate of increase in participation after implementation. The lower rates of increased participation across schools after policy implementation were based on only a few years of data, and more data may be needed to determine the association between the policy and the longer-term rate of change in breakfast participation. 

A variety of factors may have contributed to the greater observed increase in participation immediately after the UFB policy implementation among schools with lower FRP eligibility relative to those with higher. Nationally and in the state where the large urban school district is located, in the years following the Great Recession (starting during the 2007–2008 school year), participation in the school meal program increased among students who directly certified for free school meals [44,45]. This increase had leveled off prior to the UFB policy implementation in 2013–2014, so the uptick in participation immediately following the policy was likely not driven by an increase in students who qualify for free and reduced-price meals because of the Great Recession. 

However, nationally, participation among students who did not qualify for FRP meals decreased during the years following the Great Recession and continued to decrease nationally after 2013–2014 [44,45]. Trends among schools with a lower percent of FRP-eligible students in the large urban school district showed similar trends prior to 2013–2014, but unlike the national trend, there was an increase in participation among these students following the implementation of the UFB policy. Students who did not qualify for FRP meals, therefore, may have driven the increase in participation among lower FRP schools. These students, who normally might not be incentivized to eat breakfast at school due to cost and perhaps other barriers, may have found the option more appealing since breakfast was free. 

It is also possible that if more students who were not eligible for FRP meals were participating at the schools with lower FRP eligibility due to the policy, there may have been less perceived stigma around eating school breakfast, and more FRP-eligible students may have participated as well. Other studies have reported an increase in the sense of community and a reduction of stigma in schools that have implemented alternative breakfast services models and increased breakfast participation [46,47]. Unfortunately, for this study, changes to the way that FRP eligibility was determined and inconsistencies in the FRP data did not allow for direct comparisons of changes in participation among free, reduced-, and full-price students. Barriers to breakfast participation at higher-FRP schools may also differ from those at lower-FRP schools, and a UFB policy alone may not be able to address those barriers. Schools may need to implement other service models such as breakfast in the classroom to further increase participation.

As for associations between the UFB policy and attendance, there was very little change in total attendance (excused and unexcused absences combined) and percent of days tardy in schools overall before and after the policy was implemented. The small changes that were observed did not differ significantly for schools that had an increase in SBP participation relative to those that did not. There was an increase in unexcused absences on average across schools, and the percent increase in unexcused absences was slightly higher for schools that increased participation in breakfast, but this difference was not statistically significant. Individual students in schools across the district may have been on time and present more often due to the availability of free breakfast, but these changes were not large enough to detect on the school level. Some studies of universal free meal policies did observe increases in attendance among some student populations [27,30,35,48]. For example, a study of a UFB policy in New York City schools found that among 3rd to 8th graders there was a small increase in breakfast participation and a small increase in attendance for black students eligible for free meals [30]. Bartfeld et al. found that UFB was associated with an increase in the percent of days attended among low-income elementary school students in Wisconsin [48]. In addition, a study of the implementation of a UFB program in one public school in Philadelphia, PA (grades K-6) and two public schools in Baltimore, MD (grades K-8) found breakfast participation nearly doubled, and students who increased their participation had greater decreases in absences and tardiness than students whose participation remained the same or decreased [27]. However, a study of a UFB program in San Diego elementary schools found no significant change in attendance [32].

Finally, on average, there was very little change in the percent of students classified as overweight or obese in the schools included in the study between the 2012–2013 and 2013–2014 school years. Similarly, at the national and state-level, the percent overweight and obese children and adolescents also remained relatively stable during those years [49,50]. Nationally, between 2011-2014, approximately 17% of children and adolescents aged 2–19 years were considered obese [49], and the percent of students classified as overweight or obese in grades 9–12 fluctuated between 27 to 30% between 2003 and 2013 [50]. There has been some concern that UFB programs may result in excess calorie consumption among students eating more than one breakfast [51]. While it is premature to draw conclusions about the longer-term impact of the UFB policy on student weight status in the school district in this study, these early results do not provide evidence of excess calorie consumption and subsequent weight gain. Other studies of UFB programs and alternative breakfast service models have found similar results [28,35,52,53]. A three-year pilot study of the effects of a UFB program that was conducted in elementary schools in six US school districts found students’ calorie consumption over 24 hours was not affected by the availability of free breakfast, and there was no evidence of improvements in nutrition intake or health (as measured by age-adjusted BMI) [29,52]. However, students at the treatment schools were more likely to consume a substantive breakfast (a meal with food from at least two of the five food groups) and were more likely to consume more servings of fruits and dairy at breakfast than control students [53]. A longitudinal study examining breakfast consumption patterns among middle school students by location (none, home, school, both), found increased odds of overweight/obesity among frequent breakfast skippers compared to double breakfast eaters, and double breakfast eaters had weight changes that were similar to other students [54]. However, an evaluation of a free breakfast in the classroom program in New York City found that some students in elementary schools with breakfast in the classroom consumed more than one breakfast and consumed more calories in the morning than students not offered breakfast in the classroom [55]. This study only examined differences in morning calories consumed and not calories over a full day. A more recent study of the New York breakfast in the classroom program found no evidence that breakfast in the classroom increased student BMI or the incidence of obesity among students [56].

### Limitations

All schools in the large urban school district implemented the UFB policy in the same year, so it was not possible to compare changes in participation levels and other outcomes to schools that did not implement the policy. Future studies could compare trends in the outcome measures to other districts, but the size and demographic characteristics of the school district made it difficult to compare to other districts. In addition, the observational study design does not allow us to determine whether the UFB policy actually caused the changes in participation. It is possible that other factors, including the impact of the Great Recession, may have influenced participation rates.

In addition, individual student FRP eligibility and meal participation data were not available, so determining student-level associations between changes in breakfast participation and other outcomes was not possible. Additionally, changes in the percent of students classified as overweight or obese observed in this study may not be representative of all schools across the district since data were not available for all schools. Middle and high schools were more likely to not have height and weight data. However, height and weight data were available for approximately 70% of the schools in our study sample. Finally, while similar studies have examined the impact of UFB policies on test scores, due to substantial changes in the end-of-year testing, the test scores before and after the implementation of the UFB policy in the school district were not comparable.

## 5. Conclusions

Despite these limitations, this study adds to the evidence that universal free breakfast policies are associated with increases in participation in the School Breakfast Program. Past studies have focused primarily on elementary schools, but this study included schools of all grade levels, and results indicate that increases in participation after the implementation of UFB policies are also possible in middle and high schools. While some schools had very large increases in participation, upwards of 28 percentage points, other schools had more modest increases or no increase following the UFB policy. As a result, additional strategies may be needed to overcome barriers to participation in the SBP. The results do not provide evidence of excess calorie consumption and subsequent weight gain immediately following the UFB policy implementation, nor do they provide conclusive evidence about associations between UFB policies and attendance. Future studies should examine the longer-term effects of the policy on breakfast participation and other student-level outcomes. Studies could also examine the factors that led to a greater increase in breakfast participation after the implementation of the UFB policy at some schools relative to others.

## Figures and Tables

**Table 1:** Characteristics of schools that increased participation in the National School Breakfast Program and those that did not before (2012–2013) and after (2013–2014) the implementation of a districtwide universal free breakfast policy in a large urban school district in the Southeast United States.

	All Study Schools (*n* = 150)		Schools with Increase in Participation (*n* = 122)		Schools with No Increase in Participation (*n* = 28)	
	2012–2013	2013–2014	2012–2013	2013–2014	2012–2013	2013–2014
Mean Percent Breakfast Participation (SD ^a^)	24.1 (17.6)	28.9 (16.7)	20.9 (15.4)	27.6 (16.3)	38.1 (19.8)	34.5 (17.9)
Mean Percent Attendance (SD)	95.0 (1.4)	95.3 (1.5)	95.2 (1.3)	95.5 (1.5)	94.2 (1.5)	94.7 (1.4)
Mean Percent Unexcused Absences ^b^ (SD)	52.7 (13.5)	58.2 (12.2)	51.4 (13.9)	57.3 (12.6)	58.0 (10.3)	61.8 (9.8)
Mean Percent Days Tardy ^c^ (SD)	3.7 (1.6)	3.7 (2.1)	3.7 (1.6)	3.5 (2.1)	4.1 (1.8)	4.3 (2.0)
Mean Percent Students Classified as Overweight or Obese (*n*, SD)	31.8 (86, 9.7)	32.0 (86, 9.4)	30.5 (68, 9.7)	30.7 (68, 9.4)	36.8 (18, 7.9)	36.8 (18, 8.1)
Mean Percent Students of Color (SD)	70.7 (26.3)	71.3 (26.1)	67.4 (26.5)	68.1 (26.3)	85.1 (20.1)	85.0 (20.8)
Mean Percent FRP Eligible (SD)	59.8 (28.4)	60.3 (29.6)	54.4 (27.0)	55.5 (28.8)	83.4 (21.8)	81.1 (23.5)
Percent Elementary Schools	59.3	59.3	61.5	61.5	50.0	50.0
Percent Middle Schools	18.0	18.0	17.2	17.2	21.4	21.4
Percent High Schools	14.0	14.0	13.9	13.9	14.3	14.3
Percent Other Schools	8.7	8.7	7.4	7.4	14.3	14.3

^a^ Standard deviation (SD). ^b^ Unexcused absences are absences from school for which there is not an allowable excuse. The percent of unexcused absences was calculated for each school by dividing the total number of unexcused absences by the total number of absences. The mean percent unexcused absences is the average of the percent unexcused absences across schools. ^c^ Tardy refers to arriving after the start of the official school day. The percent of days tardy was calculated for each school by dividing the total days tardy by the total number of days in membership. The mean percent days tardy is the average of the percent of days tardy across schools.

## Data Availability

To request access to de-identified data, please contact the corresponding author.
